# Congenital liver hemangioma revealed by cholestasis syndrome: report of a rare case

**DOI:** 10.11604/pamj.2020.36.192.21411

**Published:** 2020-07-17

**Authors:** Chaimae Khairoun, Amina Barkat

**Affiliations:** 1 Medical Department of Neonatology Reanimation, The Reference National Centre of Neonatology and Nutrition of Mother and Child, Sick Child Hospital CHU of Rabat, Faculty of Medicine and Pharmacy, University Mohammed V, Rabat, Morocco

**Keywords:** Liver hemangioma, congenital, cholestasis, neonatal, beta-blockers

## Abstract

Our paper reports a case of hepatic angioma revealed by neonatal cholestasis, thing that has never been reported in the literature to our knowledge. A newborn boy of 25 days of life had cholestatic jaundice since his fifth day of life. During its health assessment, the angioscan detected the presence of multiple hepatic agiomas. The rest of the etiological report returned without any anomaly. Beta-blockers were started with a very good clinical and ultrasonographic evolution after 12 months of treatment.

## Introduction

Cholestasis is a discomfort in the biliary excretion. It is often accompanied by jaundice, which must always be explored if it is prolonged (beyond 10 days of life) [1]. The prevalence of neonatal cholestasis is estimated at 1/2500 [1]. This symptom must always be considered as an emergency, especially when taking in charge a biliary atresia. However, there are other rarer diagnoses, including hepatic hemangioma, that could potentially threaten patients' lives (heart failure) [1]. Neonatal cholestasis is a rare revelation of a liver hemangioma, hence the importance of this observation.

## Patient and observation

New born at 25 days of life, male, eutrophic was resulted from a badly followed full-term pregnancy. Maternal serologies were not done. The birth weight was 3000g. The APGAR scoring system was not specified with immediate scream notion. The Parents are not a consanguineous couple. Its symptomatology dates back to the fifth day of life by the appearance of a permanent cholestatic cutaneous and mucosal jaundice with completely discolored stools (white) and dark urine, without pruritus, all evolving in a context of apyrexia and conservation of the general state. This situation drived the patient to the prefectural hospital in the 24^th^ day of life. The newborn was after transferred to our university hospital centre for an etiological assessment and taking charge of cholestatic jaundice. Clinical examination at admission showed a newborn jaundice, tonic, reactive, weight = 3700g; size = 56cm; PC = 37cm; PO = 34cm; sucking reflex was present as well as archaic reflexes. He had no facial dysmorphism or externalized bleeding. Mucocutaneous examination found three punctate and superficial cutaneous angiomas in the neck, abdomen and right lower limb. Abdominal examination found hepatomegaly estimated at 5cm, without splenomegaly. The cardiovascular examination was unremarkable as well as the rest of the clinical examination. A clinical state was made, having objectified a BT rate of 163.15mg/l; BD at 118.35mg/l; IDB at 44.80mg/l. Gamma-glutamyl transferase was 214 (unit) and alkaline phosphatase 300 (unit).

**Figure 1:**
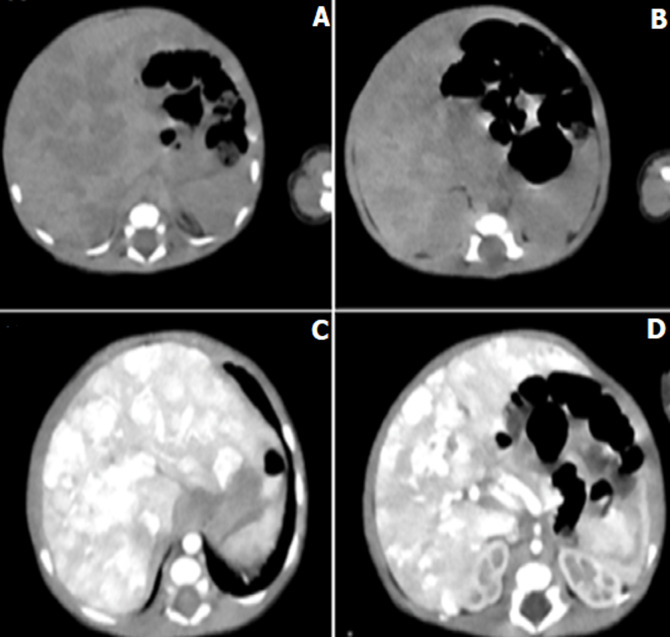
A,B) abdominal CT scan without injection; C,D) after injection showing multiple hepatic angiomas

The prothrombin rate was measured and returned to 65%. An abdominal ultrasound was also performed urgently after a strict 6-hr interval to find biliary atresia that showed hepatomegaly with hepatic mass. CBEU (cytobacteriological examination of urine) was sterile eliminating an acute pyelonephritis. Hepatitis serologies (A, B, C) were negative. The TORCH serologies were negative. The glycemic cycle in search of hypoglycaemia was normal as well as the electrophoresis of serum proteins, which showed the absence of a peak of alpha 1 globulin evoking an alpha 1 antitrypsin deficiency. In front of the ultrasound aspect, an abdominal angio-scanner was performed, which showed multiple vascular lesions (hepatic hemangiomas) with dilation of the hepatic vein, inferior vena cava and left atrium (Figure 1). Trans-fantanellar ultrasound, cervical ultrasound and reno-vesical ultrasound were performed as part of the search for other locations and returned without abnormality. Cardiac ultrasound showed a dilated left ventricle with retained function, grade II mitral insufficiency and sub-systemic pulmonary hypertension. Therapeutically, vitamin K1 was injected urgently at the dose of 2mg/kg to prevent bleeding accidents due to malabsorption of this fat-soluble vitamin. A high-calorie and high-triglyceride diet was introduced in the absence of breast milk. Beta-blockers were started (propanolol) at 2mg/kg/day twice daily with monitoring of heart rate, blood pressure and blood glucose readings, which remained normal. Attenuation of jaundice and signs of cholestasis marked the clinical course after one month of treatment. A monthly abdominal ultrasound showed the disappearance of these angiomas after 12 months of treatment.

## Discussion

Hepatic angiomas are most often fortuitous discovery following the completion of an abdominal ultrasound requested in front of a miliary hemangiomatosis or a syndrome of PHACES [2]. To our knowledge, no case of hepatic hemangioma has been revealed by a cholestasis syndrome. Diffuse forms of hepatic hemangiomas can induce heart failure because of the high rate of these angiomas. Rare cases of severe thyroid insufficiency have been reported in association with extensive hemangiomas of the liver, hypothyroidism being related to the secretion of a thyroid hormone-inactivating enzyme, 3-iodothyronine deiodinase [3,4]. In the case of our patient, the thyroid hormones were normal (TSH T3 T4) and echocardiography showed dilated left ventricle with preserved function, mitral grade II insufficiency and an infra-systemic PAH. Hepatic angioma requires prompt treatment as it can be life threatening because of high-rate heart failure [5]. Since the discovery in 2008 of their effectiveness in the treatment of infantile hemangiomas, beta-blockers have been the subject of many studies and are now the first-line treatment [6]. Propanolol has a marketing authorization (MA) for this indication. This is an oral treatment (solution) that should be started at the dose of 1mg/kg/day, taken twice daily, increased to 2mg/kg, then 3mg/kg/day to 1, then 2 weeks apart respectively [2] (Table 1). This treatment is initiated between the age of 5 weeks and 5 months according to the MA, and for a period of 6 months [7]. In the case of our patient, we used propanolol with a dose of 2mg/kg/day, with good clinical and radiological progress after twelve month of treatment.

**Table 1:** guide for the use of propranolol per os in infantile hemangiomas

When?	How?	For how long?	What to watch?
As soon as possible to avoid anatomical distortions and the development of fibro-adipose tissue, ideally before the age of 3 months.	*After elimination of a contraindication (sinus bradycardia, atrioventricular block) *Propranolol infant solution, hospital prescription (ATU), 2 to 3 mg/kg/day in 2 or 3 doses (1st week at 1mg/kg/day, then 2mg/kg/day)	*Up to 9 months of age for medium-sized hemangiomas *Up to 12 months of age, or longer in large segmental hemangiomas.	*Hypoglycemia: To avoid hypoglycaemia, make sure that the child sucks at fixed times. *Stop taking propranolol for a few days if insufficient food intake, gastroenteritis, bronchospasm during an inter-current infectious episode, and definitely if it is a recurrence or a spontaneous bronchospasm (possible revelation of an asthma).

## Conclusion

Before any neonatal cholestasis, it is always necessary to think of a congenital hepatic angioma, which belongs to the benign vascular tumours. This can put the patient's vital prognosis at risk by its cardiac repercussion requiring a quick support. Rapid treatment is now available. It is more effective if it is instituted very early: beta-blockers and in particular propranolol, a pediatric form in solution that is already available for temporary use authorization (TUA).
